# Detour test performance of cloned minipigs from three different clone populations

**DOI:** 10.1007/s11259-023-10168-0

**Published:** 2023-07-15

**Authors:** Aurora Paganelli, Martina Felici, Luca Turini, Paolo Baragli, Lucia Carlucci, Fabio Anastasio Recchia, Micaela Sgorbini

**Affiliations:** 1 Institute of Life Sciences, Scuola Superiore Sant’Anna, Piazza Martiri della Libertà, 33, Pisa, 56127 Italy; 2 Department of Agricultural and Food Science, Viale Giuseppe Fanin, 40-50, Bologna, 40127 Italy; 3 Department of Agriculture, Food and Environment, Via del Borghetto 80, Pisa, 56124 Italy; 4 Department of Veterinary Sciences, Viale delle Piagge 2, Pisa, 56122 Italy; 5 Institute of Clinical Physiology, National Research Council of Italy, Pisa, Italy

**Keywords:** Clone, Minipig, Detour test, Animal behavior

## Abstract

Genetics, the uterine environment, maternal behavior, and rearing conditions can all influence animal behavioral phenotypes. Some studies on cloned pigs have found no differences between the behavioral patterns of cloned and non-cloned animals. Other studies conducted on dogs have reported similarities in the behavior of cloned subjects. This study evaluated the performance of 12 cloned minipigs from three different clone populations (A, B, C) in a detour test around symmetric and asymmetric barriers. We measured the detour time and patterns, in order to investigate the pigs’ cognitive abilities.

The detour time and the detour entry/exit pattern were recorded. All the animals tended to keep a fixed entry/exit pattern instead of modifying it to accommodate changes in the working set. Significant differences in detour time were found among the populations, with animals belonging to population B being faster than the others, and also within each population.

Our study is one of the few to assess the cognitive abilities of cloned minipigs. The results indicate that even animals belonging to the same cloned population may develop different cognitive, hence behavioral characteristics. Whether cloning can be utilized to obtain similar behavioral phenotypes therefore remains a matter of debate.

## Introduction

Animal cloning has benefited different research areas, such as human medicine and agricultural production (Coulon et al. 2010). Animals are mainly cloned to obtain the desired phenotypes (Coulon et al. 2010). However, the expression of a phenotypic feature is determined not only by genetics, but also by cloning procedures and the environment (Archer et al. 2003; Søndergaard et al. 2012). For example, cloning procedures (Søndergaard et al. 2012), the uterine environment, maternal behavior and rearing conditions can all influence animal behavioral phenotypes beyond the behavioral characteristics of the donor animal (Archer et al. 2003; Kabadayi et al. 2018).

Some studies have thus tried to exclude certain phenotype-influencing factors in order to evaluate the effects of individual factors. The behavioral phenotypes of cloned vs. naturally bred animals have also been compared to assess the individual effects of the environment (Archer et al. 2003; Savage et al. 2003; Coulon et al. 2010; Søndergaard et al. 2012; Shin et al. 2016). However, the results are conflicting. Some studies on pigs have found no differences between the behavioral patterns of cloned and non-cloned animals (Archer et al. 2003; Søndergaard et al. 2012). Other studies conducted on dogs have reported behavioral similarities within the cloned animal group and between donor animals and naturally bred animals (Choi et al. 2014; Shin et al. 2016; Kim et al. 2018). Lastly, some studies have found a greater interaction and inter-specific recognition among cloned heifers, however no differences in behavioral variability were found compared with naturally bred animals (Savage et al. 2003; Coulon et al. 2010).

Since behavioral variability in cloned animals is still a matter of debate, some authors have used ad hoc tests to evaluate the behavior of cloned animals (Archer et al. 2003). Specifically, cloned pigs were tested for variability in temperament, taste, and object recognition compared to non-cloned conspecifics (Archer et al. 2003; Søndergaard et al. 2012). Given their great cognitive abilities in identifying food sources, and even relocated sources, tests on the feeding behavior of pigs seem promising (Nawroth et al., 2019).

The detour paradigm is often used to assess the cognitive abilities of animals. The goal of the detour test consists of reaching food as a reward after overcoming a barrier (Kabadayi et al. 2018). Through detour tests, inhibitory control, route planning ability, and learning skills can be assessed (Kabadayi et al. 2018).

In this study, the detour test was selected from among the various cognitive tests because of its versatility. We hypothesized that individual animals vary in their behavior due to the uterine environment, maternal behavior, and rearing environment in addition to genetics, and that these variations may extend to cognitive differences among individuals. Using the detour test, our study therefore aimed to assess the cognitive abilities of 12 cloned minipigs originating from three different donor animals.

## Materials and methods

### Animals

The study was conducted on 12 two-year old intact male miniature pigs, hereafter minipigs.

The mean±standard deviation weight was 31±3.63 kg, 31.4±1.89 kg and 35±2.83 kg for groups A, B and C, respectively. Three cloned populations were obtained by cloning fibroblasts from three different donor animals which were labelled Groups A, B and C, and were made up of 6, 4 and 2 animals, respectively.

**Fig. 1:**
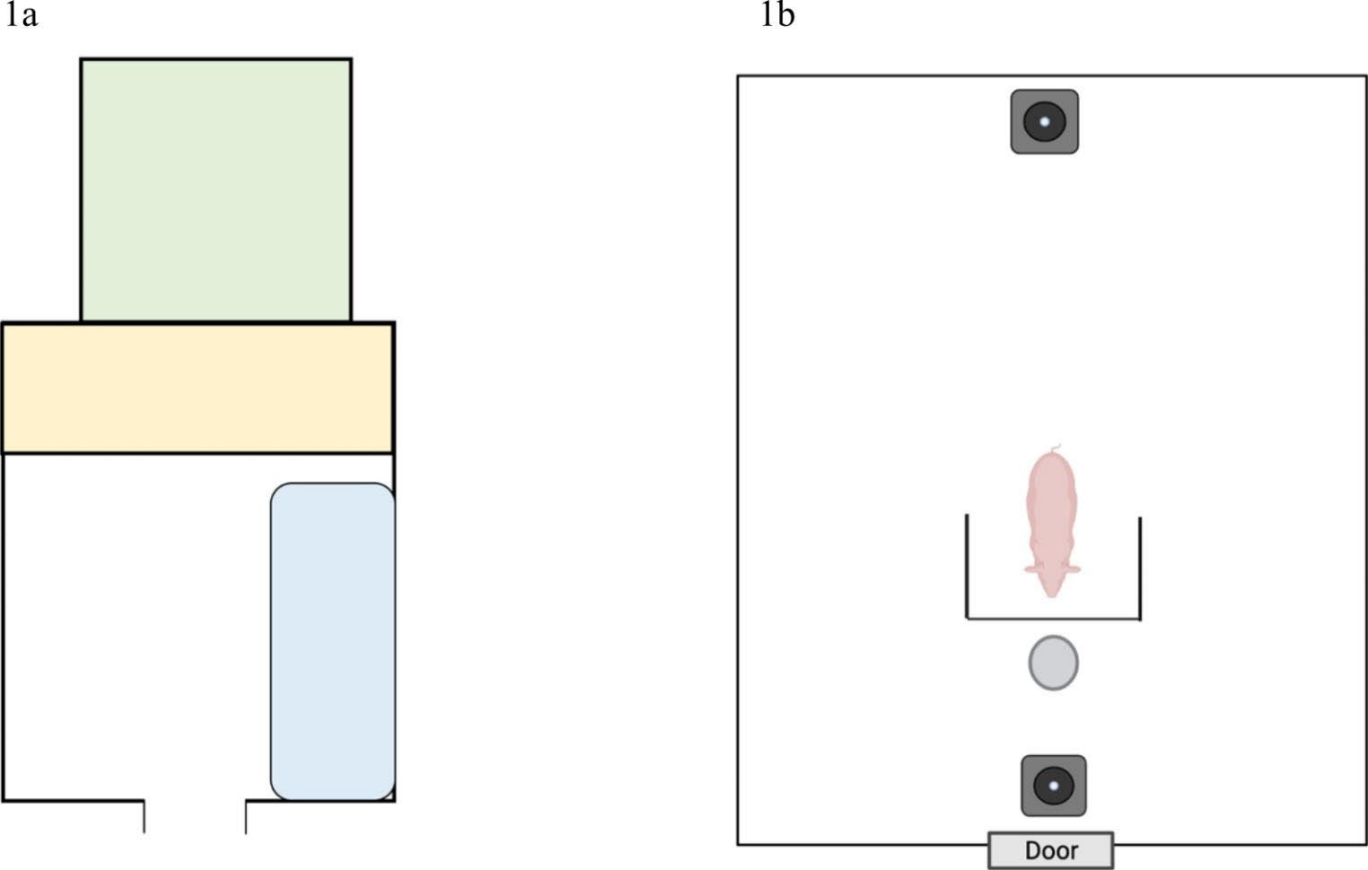
(**a**) Graphic representation of the box dedicated to the minipig’s housing. In yellow the area dedicated to rest and bedded with straw; in blue the area with nipples positioned for watering and feeders; and in green the external area closed with a 2 mt high metal fence. The inside and outside are connected by a door. (**b**) The box setting used for the detour test. Box measures 365 cm x 446 cm. This figure shows the position of the cameras (grey square with black circle inside), the position of the barrier and the position of the bowl.

The subjects were selected based on their ability to perform the detour test at the end of training sessions. All the minipigs were housed in boxes measuring 365 cm x 446 cm. Inside each box, an enriched environment was provided (metal chains, plastic balls, straw, chewable wooden sleepers), together with a rest area with a solid floor and straw bedding, and a separate feeding/walking- area. In addition to the indoor area, the minipigs could also walk in external paddocks (Fig. 1).

Animals were fed with 420 g/day of specific porcine pellets (mix of Progeo M2 pellets and Progeo Stalla fibra pellets), twice a day (8:00 am and 4:00 pm), which was administered in bowls. The animals had ad libitum access to water through drinking nipples designed for pigs. The 12 minipigs were divided into three boxes with four animals in each and were housed in homogeneous groups based on the weight and not considering the clonal line. In terms of socialization with humans, up until the time of training for this study, the animals had only come into contact with the people who took care of the housing and with the veterinarian for routine checks. They had not been subjected to specific habituation sessions in the presence of humans.

### Training/test area

For the study purposes, a single box, built as the housing box, was set up for the experimental procedures. This box had never been used to house other animals, and the minipigs were walked into it exclusively for the training sessions and then for experimental tests. Figure 1b shows the setting of the box.

**Fig. 2:**
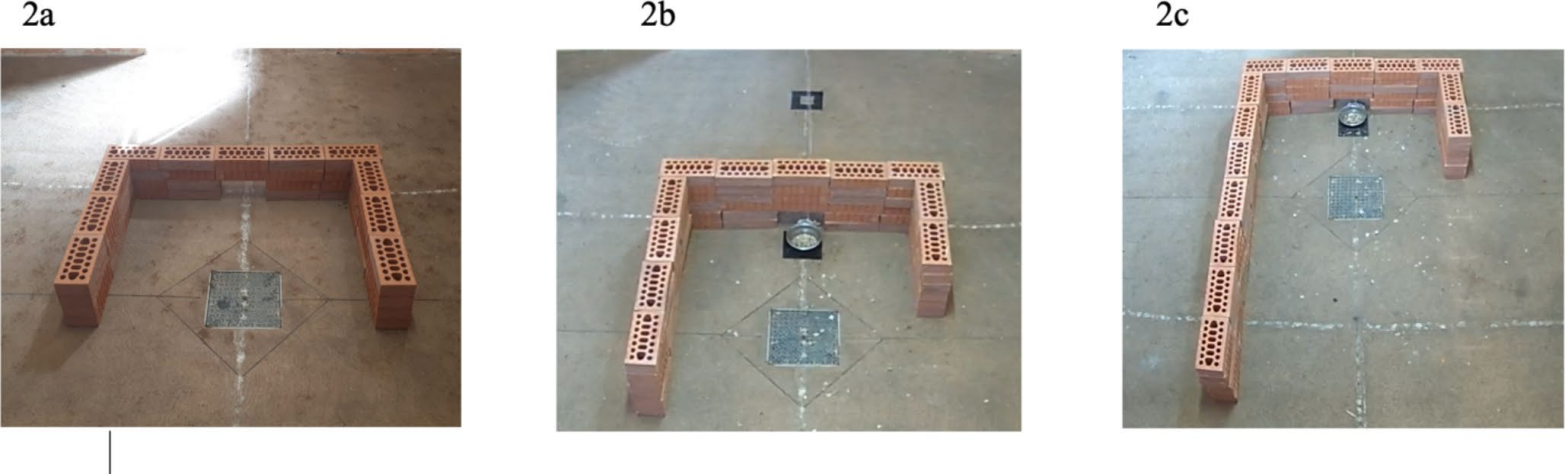
Barrier types: (**a**) symmetrical; (**b**) slightly asymmetrical; (**c**) very asymmetrical

The box setting included three types of barriers: symmetrical (SB), slightly asymmetrical (SAB), and very asymmetrical (VAB). All the barriers consisted of movable bricks, so that their shape and location could be easily changed. The height of all the barriers was approximately 15 cm (3 bricks), giving the animals no possibility of jumping over them. The SB had two equal-length lateral arms of about 60 cm each, and a front wall of about 100 cm (Fig. 2a). On the other hand, the SAB and VAB had two lateral arms that were not equal in length. The SAB had one of the two lateral arms twice as long as the others (60 cm vs. 120 cm) (Fig. 2b), while the VAB had one of the two lateral arms more than three times as long as the others (60 cm vs. 200 cm) (Fig. 2c). The front wall of the asymmetrical barriers had the same length as reported for SB. The position of the longest arm on the asymmetrical barriers was variable on the right or left side, and was chosen randomly for each training session/test repetition by tossing a coin, but the longer arm was never on the same side for more than two consecutive repetitions (Kabadayi et al. 2018). All the barriers had a little opening in the bottom center of the frontal wall enabling the operator to move the food away from the tested subject remotely at a specific time during the test. The asymmetrical barriers were only used during the experimental tests and not during the training sessions.

### Training

The minipigs underwent a period of habituation to all the procedures, locations, and materials used to conduct the detour test, and were trained in solving the detour task. For habituation, the minipigs were led one at a time into the box that had already been set up with the barrier, but without the presence of food, and were left there for a maximum of 15 min to enable them to explore freely. The training was performed using food as a positive reinforcement; the same food was also administered as a daily ration.

Before the training session the animals were administered half the food ration to keep them highly motivated during the test. The training sessions were performed on three consecutive days (Monday, Tuesday, and Wednesday), and supplementary training sessions were performed on Thursday and Friday for those animals with greater learning difficulties. The training sessions lasted no longer than 15 min, in order to maintain their motivation to search for food. If the subject did not complete the training session and lost interest in the task, it was performed again the next day.

The training session was considered successful when the pig correctly performed three consecutive repetitions of the required task (bypassing the barrier and reaching the bowl again). During the training session, the pig was directed to enter the barrier alternately, from the right or left, to prevent it from learning a preferential route before having experienced all the possibilities of entry and exit.

### Detour test procedure

The detour test evaluated the animal’s ability to bypass a barrier and reach the food on the opposite side. The food was placed in a metal bowl attached to a cord. At the beginning of the test, the bowl was placed in the inner compartment on a specific starting point marked with a “square” on the floor using adhesive tape. The minipig was spontaneously let into the box through the internal door, and then the door was quickly closed behind it. The test began when the door was closed. During the test, the operator remained outside the box, out of sight of the minipig. Once inside, the animal reached the bowl and, when the minipig began to feed, the operator, pulled the bowl away from the inner to the external area, i.e. outside the barrier which separated it from the inner compartment (Fig. 2), also marked with a “square” on the floor using adhesive tape. The test ended when the pig successfully bypassed the barrier to reach the bowl and feed again. The pig was finally let out of the box by opening the internal door.

Between the test repetitions on the same minipigs and on different minipigs, the subjects were let out of the box. The bowl full of food was then repositioned at the starting point, and then the pig were let back in. There was no disinfection between one minipig and the next.

### Experimental tests

The official tests consisted of five test repetitions for each animal carried out on three consecutive days for a total of 15 repetitions for each type of barrier used (45 repetition total) (Baragli et al. 2017). For each repetition, the animal was allowed a maximum of five minutes to bypass the barrier and reach the food. The sequence of barriers was presented always in the same way, namely SB, SAB, and VAB, with a one-week time interval in between.

During the experimental tests, detour time and scheme were recorded. The detour time was the time, measured up to tenths of a second, elapsing between feeding from the bowl placed in the area inside and outside of the barrier. The detour scheme consisted of the path followed by the minipig to enter and exit the barrier. While the evaluating operator (always the same for all the animals and all the tests) was positioned in front of the barrier, the operator’s left and right sides were defined as the “right” and “left” sides of the barrier.

### Video recording and analysis

The two cameras used for test recording were fixed above the box doors (internal door and paddock door) and positioned in front of each other to frame the barrier frontally and posteriorly, while avoiding blind spots. The paddock door was always kept closed during the tests.

Experimental tests were recorded continuously. Unnecessary parts of the recordings were deleted and the video analyses were carried out by a single operator (always the same for all the animals and all the tests). Fixed camera footage was analyzed with a VLC Media Player. Initially, the video analysis was performed at half speed, then compared to the real speed to increase the accuracy in evaluating the detour time and scheme. The video recordings were assessed by one trained experimenter. The operator who analyzed the videos was not present during the tests.

### Statistical analysis

The different sides of the minipig’s entry (right or left) or exit (right or left) from each barrier during the test was recorded. The most frequently performed pattern was considered as the main pattern which was established for each minipig. All patterns that were different from the main one were considered as variations (for example if one animal’s main pattern was right/left and in one trial it used the left/left pattern, this was considered a variation). Descriptive statistics were performed on the entry/exit patterns of the minipigs according to the different types of barriers.

Data were not normally distributed; a non-parametric analysis was thus performed. A Kruskal-Wallis analysis was used to compare the single time (i.e., the time spent by each minipig to perform a test) and the total time per day (i.e., the time spent by each minipig to perform all the repetitions of the test in a day), the detour time of the minipigs belonging to the three different groups (A, B and C), the time spent circumventing the barrier on the different days (T1, T2 and T3), the time spent circumventing the barrier according to the type of barrier used (SB, SAB, and VAB) and the interactions among these independent variables. A chi-square test was used to evaluate changes in entry/exit patterns on the different days (T1, T2, T3) and for the type of barrier used (SB, SAB, and VAB). Dunn’s post hoc test for multiple comparisons was used. Statistical analysis was performed with SAS software. Statistical significance was assigned for a P-value ≤ 0.05.

## Results

### Detour patterns

All the minipigs in the study managed to complete the training session, the SB and SAB. Repetitions and pattern changes are summarized in Table 1. Ten out of twelve minipigs (83.3%) also completed the VAB test. Therefore, Group A completed 240 repeats (two animals did not complete the VAB test), Group B completed 180 repeats, and Group C 90 repeats. The total repeats were calculated by multiplying the number of minipigs belonging to each group by the number of test repetitions performed by each animal.

**Table 1:** Repetitions and pattern changes for each experimental group. The different sides of entry (right or left) or exit (right or left) from each barrier during the test were recorded. The most frequently performed pattern was considered as the main pattern and all patterns that were different from the main one were considered as pattern changes

*Group*	*A* *n = 6*	*B* *n = 4*	*C* *n = 2*
**Total repetitions**	240	180	90
**Total pattern changes**	13	8	9
**Change in entry phase**	7	7	2
**Change in exit phase**	6	1	7

Four out of twelve minipigs (33.3%) maintained the same entry/exit pattern across all repeats, even when the barrier varied. Minipigs that never varied patterns belonged to Groups A (2/6 minipigs; 33%), C (1/4 minipigs; 25%) and B (1/4 minipigs; 25%).

Four minipigs belonging to Group A changed entry/exit patterns in 13/240 total repetitions (5.4%), 7 times when entering (2.9%) and 6 when exiting (2.5%). Three minipigs belonging to Group B changed entry/exit patterns in 8/180 repetitions (4.4%), 7 times in entry (3.9%) and once in exit (0.6%). One minipig belonging to Group C changed entry/exit patterns in 9/90 repetitions (10%), twice in entry (2.2%) and 7 times in exit (7.8%).

Changes in entry/exit patterns are summarized in Table 2. During the SB test, 6/12 minipigs (50%) changed pattern on the first day of the test. On the following days of the SB tests, 2/12 minipigs (16.7%) changed pattern on the second day, and only 1/12 minipig (8.3%) changed pattern on the third day. Regarding the SAB test, 2/12 minipigs (16.7%) showed an entry/exit pattern change on the first day of testing, while 12/12 (100%) minipigs maintained the same entry/exit pattern during the repeats on the second and the third days. Finally, during the VAB test, 3/10 minipigs (30%) changed entry/exit patterns during repeats on the first day, while 10/10 minipigs (100%) maintained the same entry/exit pattern output for all repetitions performed on the second and third days.

**Table 2:** Changes in entry/exit patterns on the three test days and for the different barrier types sorted by group. Legend: SB - symmetrical; SAB - slightly asymmetrical; VAB - very asymmetrical

*TEST*	*SB*			*SAB*			*VAB*		
GROUP	A	B	C	A	B	C	A	B	C
**DAY 1**	4	1	1	1	1	0	1	2	0
**DAY 2**	1	1	0	0	0	0	0	0	0
**DAY 3**	0	1	0	0	0	0	0	0	0

No statistically significant differences were found for the entry/exit pattern for any of the evaluation days (T1, T2, T3) and for none of the types of barriers used in this study (SB, SAB, and VAB).

### Detour time

Single detour time and total detour time are summarized in Tables 3 and 4. Both types of detour time were calculated as an average of the durations of the single detours, or the total repetitions performed by minipigs on the same day.

**Table 3:** Single detour time (seconds) for the three groups expressed as a median (minimum-maximum value), a ≠ b ≠ c, p < 0.05. *=statistical differences

*TEST*	*SB*			*SAB*			*VAB*		
GROUP	A	B	C	A	B	C	A	B	C
**ALL DAYS**	9 (6–17)	7.5 (5–14)	8.5 (6–9)	*8 (5–21)^b^	*7 (5–17)^a^	*9 (6–25)^b^	9 (5–19)^b^	6.5 (4–17)^ab^	*7.5 (5–11)^a^
**DAY 1**	*10 (8–11)^b^	8.5 (5–9)^a^	*8.5 (8–9)^ab^	8.5 (7–21)	8 (5–17)	12 (9–15)	10 (6–19)	6 (5–13)	8 (6–10)
**DAY 2**	8 (6–13)	8 (6–14)	8 (7–9)	8.5 (6–16)	10.5 (5–15)	16.5 (8–25)	7 (5–11)	5 (4–17)	7 (5–9)
**DAY 3**	*9 (6–17)^b^	6.5 (6–10)^a^	*7.5 (6–9)^b^	7.5 (5–11)	7 (6–11)	7.5 (6–9)	10 (6–16)	7 (6–8)	8.5 (6–11)

**Table 4:** Total detour time (seconds) for the three groups expressed as a median (minimum-maximum value), a ≠ b ≠ c, p < 0.05

*TEST*	*SB*			*SAB*			*VAB*		
GROUP	A	B	C	A	B	C	A	B	C
**ALL DAYS**	44 (30–66)	39.5 (26–50)	46 (36–59)	43 (28–84)	33 (23–72)	46 (32–110)	38 (29–93)	35.5 (28–63)	33.5 (26–42)
**DAY 1**	49.5 (39–58)	40.5 (37–50)	47.5 (45–50)	47.5 (35–64)	42 (27–72)	64.5 (53–76)	40 (31–93)	33.5 (30–63)	40.5 (39–42)
**DAY 2**	42.5 (30–66)	40 (34–44)	43 (39–47)	44 (36–84)	42 (30–69)	74.5 (39–110)	35 (29–67)	37 (30–54)	30.5 (26–35)
**DAY 3**	40 (31–50)	33.5 (26–43)	47.5 (36–59)	38.5 (28–55)	33 (23–44)	34 (32–36)	39 (30–82)	35.5 (28–45)	32 (32–32)

For the single detour time during the SB test, Group B were faster than Group A on day 1 (p < 0.05), and then Group A and C on day 3 (p < 0.05).

For the single detour time during the SAB test, Group B were faster than Groups A and C (p < 0.01), on all three days of the tests, while Group C was faster than Group A for the single detour time of the VAB (p < 0.05).

For the total detour time, Group B were faster than Groups A and C (p < 0.05), respectively. No differences were found with respect to the type of barrier used.

Regarding the intra-group comparison, three minipigs in Groups A and one in Group B were faster than the others belonging the same respective groups in performing tests for all the types of barriers used and for all evaluation days (p < 0.001).

## Discussion

Our aim was to investigate the cognitive abilities of 12 cloned minipigs belonging to three different populations (indicated as groups in our experiments) of donor animals using a detour test. Within the same populations of clones, the minipigs were homogeneous from a genetic point of view and managed in a similar way as regards housing and feeding. The minipigs tended to fix individual entry/exit patterns during the first day of the detour test, and the patterns remained constant during the subsequent repetitions. In fact, as the test days progressed, the number of entry/exit pattern changes decreased.

The animals fixed a pattern after a few repetitions of the test without considering the most convenient path (i.e., the shorter one) to reach the food reward. This was especially evident in the SAB and VAB tests during which the minipigs walked along the same path without considering the different lengths of the barrier arms.

This phenomenon of behavioral fixity or functional fixedness has been observed in several studies conducted on different species (Pongracz et al. 2003a, b; Osthaus et al. 2010). Functional fixedness is a cognitive constraint that may enhance the speed at which certain tasks can be accomplished (Trane et al., 2021), however it does not consider the advantage of maintaining certain behavioral patterns. Baragli and others (2017) attribute the behavioral fixity or flexibility during the performance of spatial tasks to the activation of different brain hemispheres and the manifestation of different personality traits. The fact that the behavioral fixity we found in the cloned minipigs has also been found in other naturally-bred animal species suggests that individual personality develops in response to not only genetic, but also epigenetic factors (Archer et al. 2003).

No differences were found in barrier entry/exit patterns for either the intra- and inter-group comparison. Detour times were statistically different between different populations and even within the same population. The detour time (both single and total) tended to decrease over the duration of the protocol. In fact, in all of the three trials with different barriers, the animals took less time to circumvent the barriers on day three than on day one.

This improvement in execution times highlights the presence of learning phenomena. In most studies based on detour tests, individuals are tested repeatedly. Several studies found improvements in both execution time and execution accuracy on subsequent repetitions (Wallis et al. 2001; Parker et al. 2005, 2012; Smith and Litchfield 2010; Vlamings et al. 2010; Boogert et al. 2011; Vernouillet et al. 2016). Learning is an example of phenotypic plasticity (Dukas 2004), therefore the minipigs in our study demonstrated an evolution of their behaviors based on task learning.

The fact that the entry/exit pattern did not change, and the detour times tended to decrease highlights the tendency of these animals to fix a certain behavior and create cognitive shortcuts which enabled them to speed-up decision-making (Kabadayi et al. 2018).

In our study, both intergroup (B vs. A and C) and intragroup differences were found. Minipigs belonging to the three different populations behaved differently as regards detour times. These data could be partially explained by different genetics (three different donor animals), which could have influenced the cognitive abilities of the three different groups, as previously reported (Archer et al. 2003). However, differences were also found between individuals belonging to the same population of clones.

Our results contrast with the findings by Shin et al. (2016), who showed that six cloned dogs responded similarly when subjected to a Y-maze detour test (Shin et al. 2016). This difference could be due to the different tests proposed. In Shin’s study. dogs were subjected to a Y-maze test that provided only one choice to arrive at the reward, while in our study the detour test involved both choices leading to a reward. Our findings seem to agree with the results obtained by Archer et al. (2003), who found statistically significant differences within the same population of cloned pigs, in terms of both personality and food preferences.

Our findings partially support our initial hypothesis that both genetic and epigenetic factors influence animals’ behavior and cognitive abilities. In fact, we found both intergroup and intragroup differences comparing three different groups of cloned minipigs derived from three different donor animals. These findings suggest that, in addition to genetics, the environment is important factor in terms of its influence on animal behavior. Moreover, the effects of epigenetic factors should also be considered in nuclear transfer cloning for the reproduction of animals with specific behavioral characteristics.

Some study limitations should be acknowledged. Firstly, the group sample sizes were small and not homogeneous. and the minipigs were all male. These characteristics could have affected our observations and limited the statistical power. The difficulty of obtaining cloned minipigs needs to be considered and, consequently, the rarity of experimental protocols carried out in this animal model. Secondly, we only used the detour test for the assessment of learning and cognitive abilities. Future studies should assess the individual personality of cloned animals by applying further tests. Finally, videos were assessed by one operator, and thus the inter-rater reliability was not performed.

In conclusion, this research adds to the findings shown by other authors regarding the cognitive patterns in animals. The results suggest that even cloned animals show different cognitive characteristics that lead to different behaviors. This may provide interesting insights in the multiplicity of factors that influence the development of animal behavior and indicates that, compared with other phenotypic characteristics, behavior is more difficult to reproduce by cloning.
